# EZH2 Inhibition in Mesothelioma Cells Increases the Release of Extracellular Vesicles That Skew Neutrophils Toward a Protumor Phenotype

**DOI:** 10.3390/ijms262110328

**Published:** 2025-10-23

**Authors:** Giulia Pinton, Elia Bari, Silvia Fallarini, Valentina Gigliotti, Veronica De Giorgis, Fausto Chiazza, Maria Luisa Torre, Marcello Manfredi, Laura Moro

**Affiliations:** 1 Department of Pharmaceutical Sciences, University of Piemonte Orientale (UPO), 28100 Novara, Italylaura.moro@uniupo.it (L.M.); 2 Department of Translational Medicine, University of Piemonte Orientale (UPO), 28100 Novara, Italymarcello.manfredi@uniupo.it (M.M.)

**Keywords:** EZH2 inhibition, tazemetostat (EPZ-6438), pleural mesothelioma, extracellular vesicles, neutrophils, MSLN and PD-L1 expression

## Abstract

We previously demonstrated that in BAP1-proficient pleural mesothelioma cells, *CDKN2A* is critical for mediating the response to selective EZH2 inhibition and highlighted a complex interplay between epigenetic regulation and the tumor immune microenvironment. In this study, we employed a quantitative proteomic mass spectrometry approach to assess alterations in protein expression following EZH2 inhibition in BAP1- and CDKN2A-proficient mesothelioma cells cultured as spheroids. Additionally, we analyzed extracellular vesicles (EVs), which were isolated through tangential flow filtration. Flow cytometric analysis and co-culture systems were used to characterize the effects of EVs on neutrophils. Upon EZH2 inhibition, we demonstrated RAB27b and CD63 upregulation and increased release of extracellular vesicles. We found that a brief exposure to EVs derived from EZH2 inhibitor-treated cells skewed naïve neutrophils toward a pro-tumor phenotype characterized by high levels of PD-L1 and MSLN (Mesothelin) expression on the surface. These EV-elicited neutrophils suppressed T cell proliferation while enhancing tumor cell growth. Moreover, we observed changes in the EV cargo derived from EZH2 inhibitor-treated spheroids. Our findings highlight the significant role of EVs in creating an immunosuppressive microenvironment, and underscore the urgent need for further investigation into the regulation of neutrophil biology and function in the PM.

## 1. Introduction

Pleural mesothelioma (PM) is a deadly cancer of the pleural surface that is caused primarily by asbestos fiber inhalation. Despite the banning of asbestos in many countries, an increase in PM cases is expected due to exposure in the past and continued use in developing countries [1]. Unfortunately, treatment options for PM are limited, with systemic therapy, for those patients who are not candidates for surgery, typically consisting of pemetrexed with cisplatin or carboplatin [2,3]. Recently, the combination of ipilimumab plus nivolumab has been approved as a first-line treatment for PM, offering a new option for patients [2,4]. Additionally, in September 2024, the Food and Drug Administration approved pembrolizumab with chemotherapy as a first-line treatment for unresectable, advanced or metastatic PM [5]. The effectiveness of immune checkpoint inhibitor therapy for the epithelioid subtype of PM is still uncertain, and much has to be gained to overcome the immune resistance observed in a large PM population [6]. In this context, it is crucial to identify reliable predictors of response to determine which patients would benefit most from standard chemotherapy, doublet immunotherapy, or chemoimmunotherapy. Various biological and molecular factors, including histological subtype, tumor mutational burden, and programmed death ligand 1 (PD-L1) expression, have been investigated for this purpose, but the results have been inconclusive [7,8,9,10]. Furthermore, despite remarkable advancements in the treatment of PM, patients often experience progression and relapse within six months of initiating therapy, underscoring the necessity for improved second-line treatments [11].

A recent open-label, single-arm phase 2 study investigated the efficacy and safety of tazemetostat (EPZ-6438), a selective oral inhibitor of enhancer of zeste homolog 2 (EZH2), in patients with relapsed or refractory PM characterized by BRCA1-associated protein (BAP1) inactivation [12]. Although, the response rate was modest in the phase 2 study, the drug demonstrated a more favorable adverse event profile and prolonged disease control than conventional second-line therapies. EZH2 is a core component of PRC2 (Polycomb repressive complex 2), which catalyzes the trimethylation of histone 3 on lysine 27 (H3K27me3), leading to gene repression and alterations in chromatin structure [13]. In our previous publications, we investigated the response of BAP1 wild-type PM cells to EZH2 inhibition, prompted by our observation of intense H3K27me3 immunostaining in BAP1 wild-type PM biopsies. We found that treatment with EPZ-6438 in BAP1 wild-type PM multicellular spheroids induced cell cycle arrest and increased expression of the senescence marker cyclin-dependent kinase inhibitor 2A (*CDKN2A*) [14]. Additionally, this treatment enhanced the recruitment of monocyte-derived tumor-associated macrophages (Mo-TAMs) and promoted their pro-tumor phenotype [15].

Senescent cells communicate with neighboring cells and the surrounding microenvironment by secreting bioactive molecules, which is referred to as the senescence-associated secretory phenotype (SASP) [16], and recent research has highlighted the role of small extracellular vesicles (EVs) as key components of the SASP [17]. EVs, particularly exosomes, are lipid membrane vesicles (30–150 nm in size) released from tumor cells to transfer signaling molecules [18,19]. Emerging evidence indicates that tumor-derived EVs can modulate immune cells, enabling tumor cells to evade immune surveillance [20,21]. Preclinical and clinical studies also suggest that PD-L1 expression on EVs predicts a low response rate to anti-PD-L1/PD-1 therapy [22,23]. Tumor-derived EVs utilize various mechanisms to promote immune suppression. They can directly transport mRNAs, proteins, or regulatory microRNAs, thereby influencing PD-L1 gene expression and modifying PD-L1 protein levels in both tumor and immune cells. For example, tumor-derived EVs can induce PD-L1 expression on neutrophils, subsequently leading to the inhibition of T-cell activation, proliferation, and function [24].

Neutrophils are crucial immune cells in the tumor microenvironment (TME) that perform both antitumor and protumor functions [25]. Recent research has indicated that neutrophil subsets in cancer do not exist in a binary classification but rather represent a continuum of states [26]. A high neutrophil-to-lymphocyte ratio (NLR) has been described as an independent predictor of poor survival in PM patients. The hypothesis behind this is that activated T cells might be suppressed by marked neutrophil infiltration [27]. Thus, further research is needed to better understand how neutrophils activated by tumor-derived EVs could contribute to PM progression.

In this study, we treated PM multicellular spheroids with EPZ-6438, which resulted in increased release of EVs, as suggested by proteomic analysis; neutrophils were then treated with EVs, and polarization toward a protumor phenotype was investigated.

## 2. Results

### 2.1. EPZ-6438 Treatment Induced RAB27b and CD63 Expression in MSTO-211H Spheroids, as Evidenced by Quantitative Proteomic Analysis

As previously described [14], treatment of MSTO-211H PM cells cultured as multicellular spheroids (MCSs) with EPZ-6438 led to the suppression of the trimethylation of histone H3 on lysine K27 (H3K27me3), as well as a 30% reduction in both the size of the MCSs and the number of viable cells (Figure S1).

**Figure 1:**
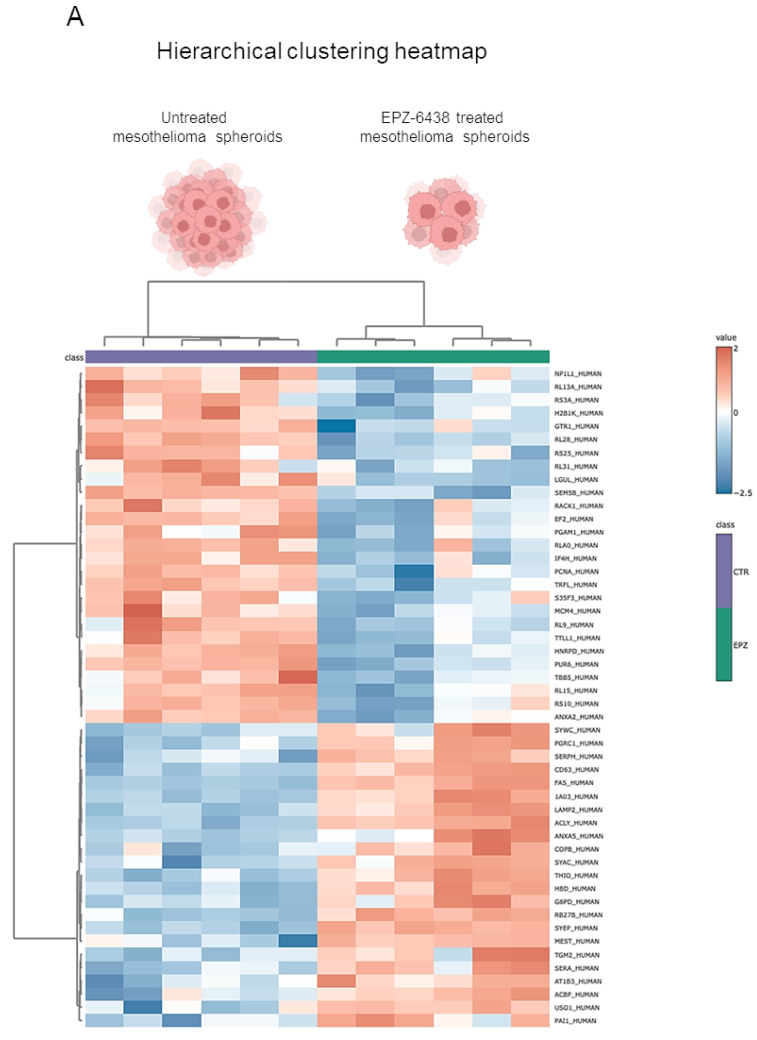

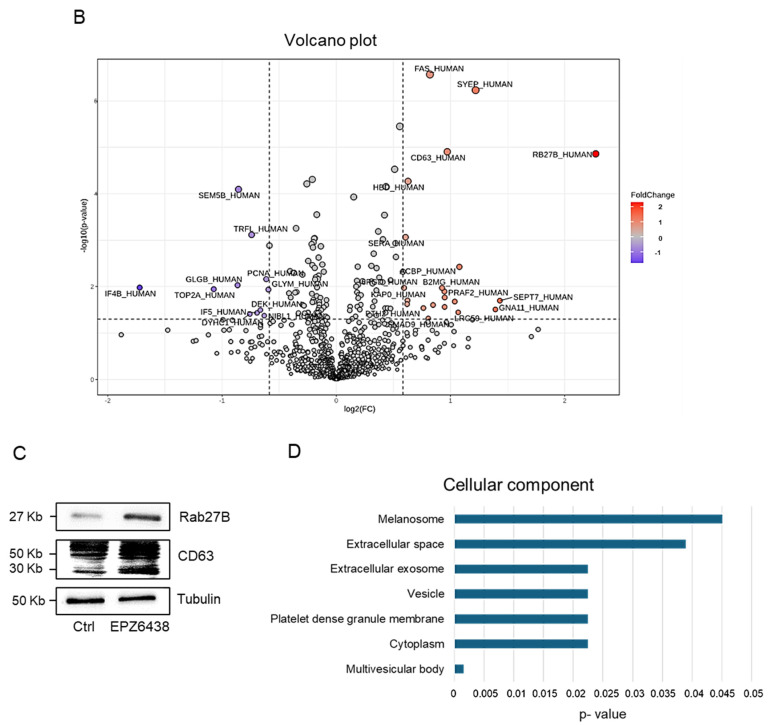
Quantitative proteomic analysis of EPZ-6438 treated MSTO-211H spheroids. (**A**) Heatmap including all 881 proteins quantified via proteomic analysis; samples treated with EPZ-6438 were separated from each other and from the control group. (**B**) Volcano plot of differential protein expression. The abscissa is the difference (linear fold change [FC] in protein concentration (logarithmic transformation with base 2), and the ordinate is the statistical significance, that is, the logarithmic transformation with base 10 for the *p* value. Red and purple dots in the volcano plot indicate proteins with significant differences (purple, *p* < 0.05 and FC < 0.67; red, *p* < 0.05 and FC > 1.5); black dots are proteins without significant changes. (**C**) Representative Western blot analysis of CD63 and RAB27b in MSTO-211H cells cultured as MCSs ± 72 h treatment with EPZ-6438. Tubulin was used as the loading control. (**D**) Gene Ontology (GO) cellular component analysis of the significantly upregulated proteins. Statistically significant categories (*p* < 0.05) are displayed in blue.

In this study, we conducted a proteomic analysis of proteins extracted from MSTO-211H MCSs cultured for 72 h in the presence or absence of 10 μM EPZ-6438. A total of 881 proteins were identified and quantified. The hierarchical clustering heatmap clearly highlighted the impact of EPZ-6438 treatment on the proteome (Figure 1A). The statistical analysis, performed with a fold change (FC) > 1.5 or <0.67 as the cutoff ratio and a *p* value < 0.05, revealed 33 differentially expressed proteins, of which 21 were upregulated and 12 were downregulated by EPZ-6438 treatment (Figure 1B).

Among the most strongly upregulated proteins in EPZ-6438-treated MCSs, we noticed RAB27b (FC = 4.8, *p* = 1.3 × 10^−5^) and CD63 (FC = 2, *p* = 1.2 × 10^−5^). The Rab GTPases play crucial roles in various stages of membrane trafficking, including the generation and release of EVs. Additionally, the exosome tetraspanin marker CD63 is positively correlated with EV secretion. The increased expression of RAB27b and CD63 was validated by Western blot analysis of lysates obtained from untreated or EPZ-6438-treated PM MCSs (Figure 1C).Gene Ontology (GO) cellular component analysis of the significantly upregulated proteins indicated associations with the melanosome, extracellular space, extracellular exosome, and vesicle (Figure 1D). This prompted us to investigate the potential impact of treating PM cells with EPZ-6438 on the release of EVs.

### 2.2. EPZ-6438 Treatment of MSTO-211H Spheroids Resulted in a Doubling of Extracellular Vesicle Release

**Figure 2:**
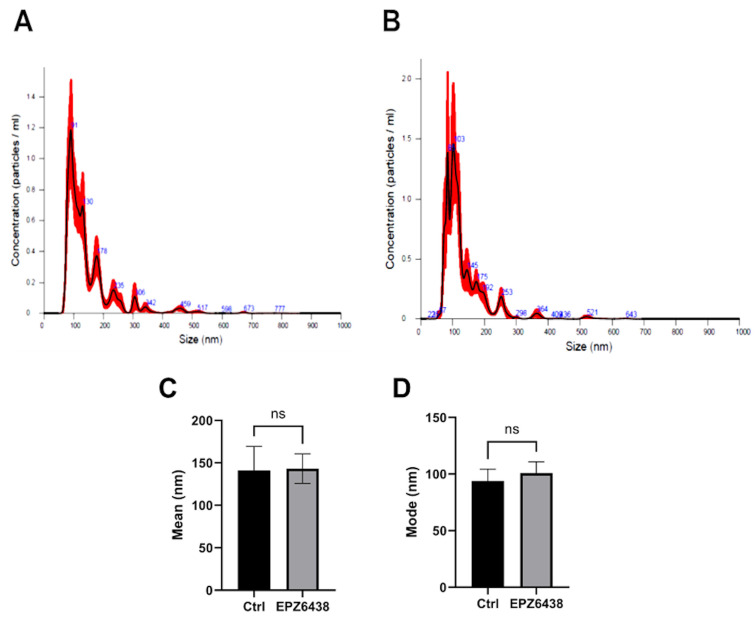

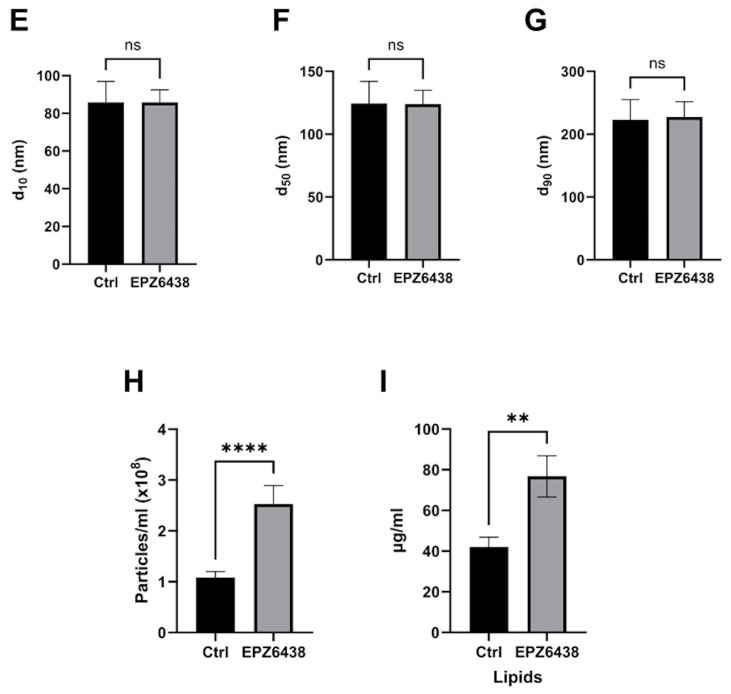
Analysis of extracellular vesicles released by MSTO-211H spheroids. Representative NTA average curves of EVs from MSTO-211H cells cultured as MCSs after 72 h of treatment in the absence (**A**) or presence (**B**) of EPZ-6438, analyzed with a NanoSight NS300 instrument: particles/mL on the vertical axis and size in nanometers (nm) on the horizontal axis. The red shadow along the line shows the dispersion of the data obtained for that sample. (**C**–**G**) Diameter percentiles (d10, d50, d90) of EVs from MSTO-211H-treated MCSs after 72 h in the absence or presence of EPZ-6438. (**H**) Concentrations of EVs from MSTO-211H cells cultured as MCSs ± 72 h after treatment with EPZ-6438 were analyzed with a NanoSight NS300 instrument. The amount of particles/mL was normalized to the number of cells. (**I**) The bar graph shows the relative total lipid content analyzed with Nile red in EVs from MSTO-211H cells cultured as MCSs ± 72 h treatment with EPZ-6438. The μg of lipids per mL was normalized to the number of cells. Each bar represents the mean of three independent experiments ± SD, ** *p* ≤ 0.01, **** *p* ≤ 0.001, *ns*: not significant.

MSTO-211H spheroids were formed through forced aggregation in non-adherent 96-well microplates in complete medium for 24 h; subsequently, the medium was replaced with serum-free medium containing or not containing 10 μM EPZ-6438. Conditioned media from both control and treated MCSs were then collected twice daily for three days, and EVs were isolated via ultrafiltration. Both EV batches were then freeze-dried to obtain a ready-to-use powder product for the subsequent experiments. The diameter of EVs released by PM MCSs treated with EPZ-6438 or not matched the typical size of EVs (Figure 2A,B). The mean size of the EVs from untreated PM MCSs was 145.4 ± 4.6 nm, whereas that from EPZ-6438-treated PM MCSs was 133.8 ± 4.8 nm. Nanotracking profiles revealed no significant differences in the mean diameter, mode, d_10_, d_50,_ or d_90_ of EVs from PM MCSs grown with or without EPZ-6438 (Figure 2C–G), but the EV content, normalized by cell number, was more than double (*p* < 0.00001) for PM MCSs treated with EPZ-6438 when determined with NTA (Figure 2H). These values were confirmed with Nile red staining (Figure 2I).

### 2.3. Extracellular Vesicles Derived from EPZ-6438-Treated MSTO-211H Spheroids Induced PD-L1 Expression on Neutrophils and Enhanced Their Immunosuppressive Functions

Numerous studies have shown that EVs released from tumor cells function as significant mediators of immune regulation in cancer [28]. Recent evidence indicates that neutrophils, a key component of the inflammatory tumor microenvironment (TME), possess a high degree of plasticity in response to various environmental signals, including EVs [29].

In our study, we analyzed the effects of incubating a set of naïve neutrophils obtained from healthy donors with EVs released from MSTO-211H MCSs treated with or without EPZ-6438. The light microscope images showed increased aggregation of neutrophils upon 3 h of incubation with EVs from PM MCSs treated with EPZ-6438 (Figure S2). We excluded a direct effect of EPZ-6438 on neutrophils based on Western blot analysis, which showed no changes in H3K27me3 levels following treatment (Figure S3).

**Figure 3:**
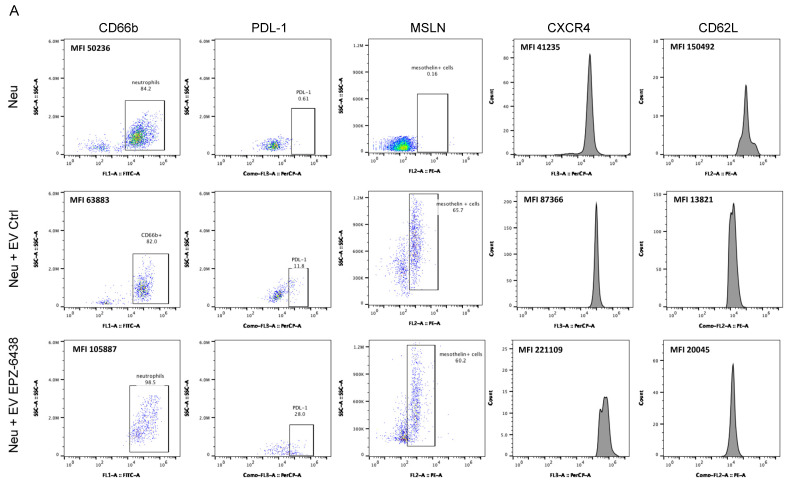

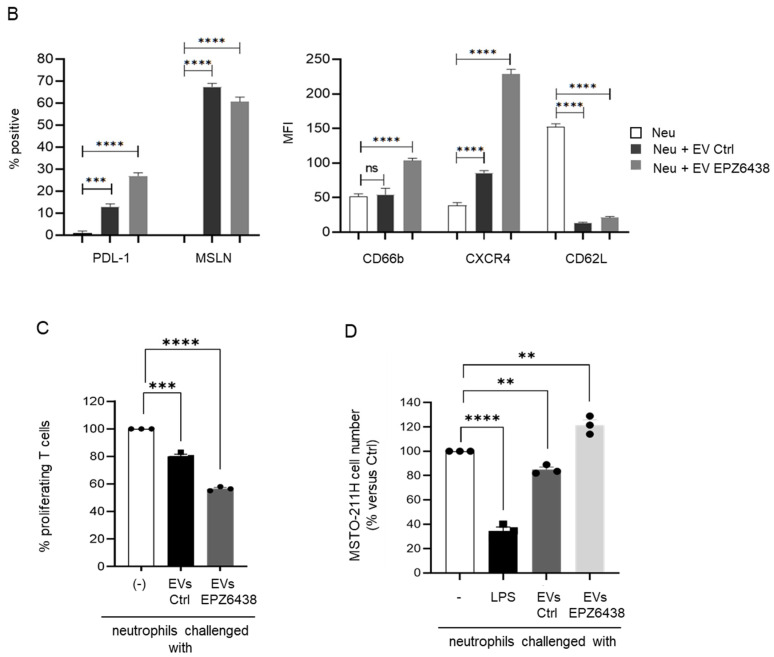
Analysis of neutrophils challenged with EVs from MSTO211H spheroids. (**A**,**B**) Representative plots and histograms from FACS analysis, and bar graphs showing the percentage of positivity of PDL-1 and MSLN, and the mean fluorescence intensity (MFI) of CD66b, CXCR4, and CD62L on the surface of neutrophils incubated for 3 h with EVs isolated from MSTO-211H MCSs ± 72 h of treatment with EPZ-6438. (**C**) Bar graph showing the percentage of proliferating PMA-stimulated T cells after 24 h of incubation with naïve neutrophils or challenge with EVs from untreated or EPZ-6438-treated MSTO-211H MCSs. (**D**) Bar graph showing the number of MSTO-211H MCSs incubated for 24 h with naïve neutrophils or challenged with EVs from untreated or EPZ-6438-treated MSTO-211H MCSs, expressed as the percentage of untreated MCSs. The histograms in (**A**–**C**), show the means ± SD, ** *p* ≤ 0.01, *** *p* ≤ 0.005, **** *p* ≤ 0.001, *ns:* not significant.

Then, we analyzed the expression of a panel of neutrophil-specific molecules in neutrophils challenged with or without EVs via flow cytometric analysis. Interestingly, flow cytometric analysis revealed that approximately 60% of neutrophils treated with EVs from PM cells expressed on their surface MSLN (Mesothelin), a sensitive marker for PM. These findings support the idea that direct membrane fusion is one of the mechanisms of tumoral EV uptake in neutrophils (Figure 3A,B). EVs collected from untreated PM cells led to increased expression of PD-L1 (Programmed Death-Ligand 1) and CXCR4 (C-X-C chemokine receptor type 4) on neutrophil membranes, along with decreased expression of L-selectin, also known as CD62L (Figure 3A,B). EVs from EPZ-6438-treated PM cells caused increased expression of activation marker CD66b, and even significantly increased expression of PDL-1 and CXCR4 on neutrophil membranes (Figure 3A,B).

A recent study reported that a subset of PD-L1+ tumor-associated neutrophils (TANs) can directly suppress T-cell-mediated immunity [22]. We cocultured neutrophils challenged with EVs from untreated and EPZ-6438-treated PM MCSs with PHA-activated T-cells derived from the peripheral blood of healthy donors. Interestingly, neutrophils challenged with EVs from PM cells significantly suppressed T-cell proliferation, and more significantly, those challenged with EVs released from EPZ-6438-treated cells did (Figure 3C).

Conversely, when neutrophils challenged with EVs were cocultured with PM MCSs, they promoted cell growth (Figure 3D). LPS (lipopolysaccharide) was used as a control to polarize neutrophils toward an antitumor cytotoxic phenotype. Collectively, these results indicate that neutrophils activated by PM-derived EVs, particularly those derived from EPZ-6438-treated cells, play a key role in inducing PD-L1 expression and promoting immunosuppressive functions in neutrophils. 

### 2.4. Proteomic Analysis Identified Differentially Regulated Proteins in Extracellular Vesicles Derived from EPZ-6438-Treated MSTO-211H Spheroids

**Figure 4:**
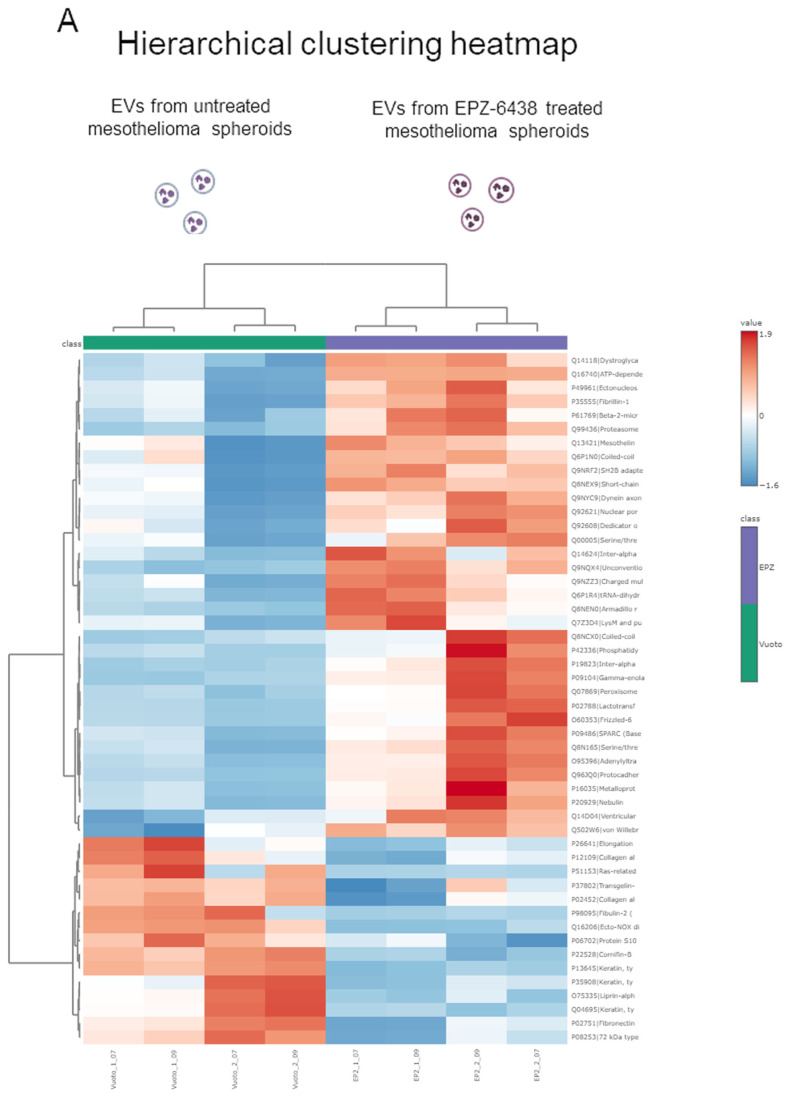

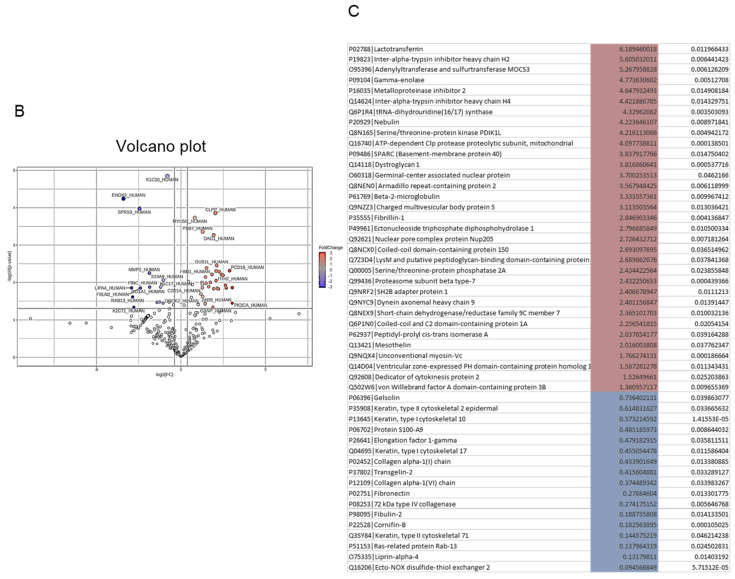
Proteomic analysis of EVs derived from MSTO211H spheroids. (**A**) Heatmap including proteins quantified via proteomic analysis; EVs from MSTO-211H MCSs treated with EPZ-6438 were separated from each other and from the control group. (**B**) Volcano plot of differential protein expression. The abscissa is the difference (linear fold change [FC] in protein concentration (logarithmic transformation with base 2), and the ordinate is the statistical significance, that is, the logarithmic transformation with base 10 for the *p* value. Red and purple dots in the volcano plot indicate proteins with significant differences (purple, *p* < 0.05 and FC < 0.76; red, *p* < 0.05 and FC > 1.3); black dots are proteins without significant changes. (**C**) List of the 37 more concentrated (red) and the 17 less concentrated (blue) proteins in EVs from MSTO-211H MCSs treated for 72 h with EPZ-6438 than in those from untreated MCSs.

We compared the protein cargo of EVs released by MSTO-211H MCSs treated or not with EPZ-6438. As shown by the hierarchical clustering heatmap (Figure 4A), the proteome profiles of EVs from treated and untreated MCSs were very different. A total of 391 proteins were identified, and 54 differentially expressed proteins were detected (fold change (FC) > 1.3 or <0.76 and *p* value < 0.05), as reported by the volcano plot in Figure 4B, of which 37 were more concentrated and 17 were less concentrated in EVs from EPZ-6438-treated MCSs than in those from untreated MCSs (Figure 4C). Frizzled 6, phosphatidylinositol 4,5-bisphosphate 3-kinase catalytic subunit alpha and peroxisome proliferator-activated receptor alpha were the most upregulated proteins, whereas the most downregulated protein was Ecto-NOX disulfide-thiol exchanger 2. Interestingly, among the enriched proteins in the EV cargo, while MSLN (FC = 2) was expressed on the neutrophil surface after treatment with EVs from PM MCSs, we did not identify PD-L1.

## 3. Discussion

Owing to its significant role in tumor initiation and progression, targeting EZH2 has become a crucial therapeutic strategy in the treatment of a variety of cancers [30,31]. Several EZH2 inhibitors have been developed, including tazemetostat (EPZ-6438), which has received FDA approval for the treatment of follicular lymphoma and epithelioid sarcoma [32]. Research on combining EZH2 inhibitors with immune checkpoint blockade (ICB) treatment may guide the development of future combination therapies [33]. The significant epigenetic and transcriptomic alterations caused by EZH2 in both tumor and immune cells play a key role in shaping the immune-suppressive activity of solid tumors [34].

We recently reported that treatment of PM spheroids with EPZ-6438 enhanced both the recruitment of monocyte-derived tumor-associated macrophages (Mo-TAMs) and the expression of their protumor phenotype [12]. Thus, exploring the distinct changes in the tumor microenvironment (TME) triggered by EZH2 inhibition could provide valuable insights for strategically combining different immunotherapies.

Malignant cells can evade immune surveillance through various mechanisms. One well-known mechanism is the interaction between PD-L1 and PD-1, which contributes to immunosuppression [35]. Studies have shown that infiltrating neutrophils can express high levels of PD-L1, leading to the suppression of T-cell activation and proliferation [36]. The complex network of interactions between tumor cells and distinct non-tumor cells is influenced by numerous factors. Among other factors, cellular communication through the release of EVs plays a crucial role in cancer development and maintenance [37,38]. Tumor-derived EVs interact with healthy neighboring cells, promoting tumor growth and expansion; for example, the interaction between tumor cells and fibroblasts, as well as between endothelial, mesenchymal, and immune cells, is well documented [39]. Understanding this mode of cellular communication is essential for uncovering biological processes and advancing diagnostic and therapeutic strategies for targeting diseases, thus improving patient outcomes.

**Figure 5:**
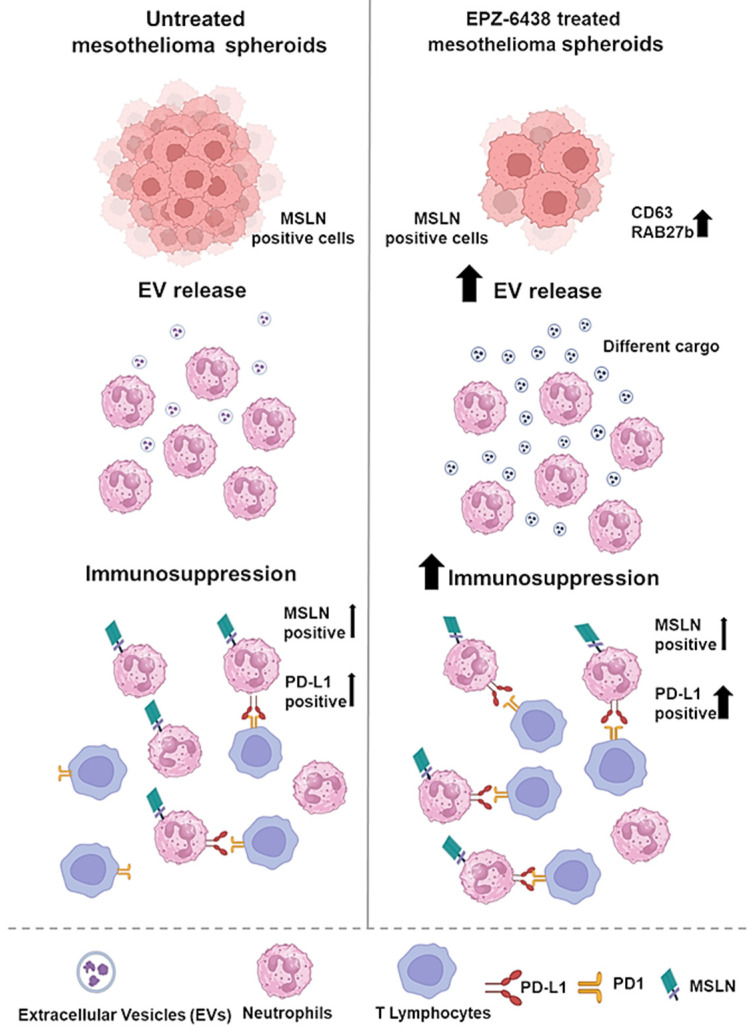
Graphical summary of the experimental data presented.

Proteomic analysis of PM spheroids treated with EPZ-6438 revealed a significant increase in the expression of RAB27b and CD63, indicating increased release of EVs. Subsequent purification and analysis of EVs confirmed these findings. Here, we present initial evidence that EVs derived from PM cells, particularly those derived from EPZ-6438-treated cells, induce PD-L1 expression in neutrophils. Our study is the first to demonstrate that PM-EVs trigger the development of immunosuppressive neutrophils, leading to the inhibition of T-cell immunity (Figure 5). These results highlight the significant role of EVs in creating an immunosuppressive microenvironment, shedding light on the regulation of neutrophil biology and function in the PM.

The molecular mechanisms underlying EV-mediated upregulation of PD-L1 expression appear to be complex. Studies have shown that EVs can transport PD-L1 mRNA directly to increase its transcription in tumor cells [40]. Additionally, exosomal noncoding RNAs have been implicated in the upregulation of PD-L1 expression in macrophages and monocytes [41]. Moreover, metastatic melanoma, breast cancer, and head and neck squamous cell carcinoma (HNSCC) cells release EVs containing the PD-L1 protein on their surface [42]. This PD-L1 protein can directly inhibit the killing of tumor cells by T-cells and promote tumor growth.

Recent findings indicate that the PI3K/AKT signaling pathway is involved in the modulation of PD-L1 expression in melanoma cells and may play a part in PD-L1-mediated immune-independent resistance [43]. In addition, M2-type tumor-associated macrophages (TAMs) can increase PD-L1 expression in cervical cancer cells via the PI3K/AKT pathway, increasing their migration and invasion capabilities and affecting tumor progression [44,45]. Notably, proteomic profiling of the cargo of EVs isolated from EPZ-6438-treated PM MCSs revealed that the alpha catalytic subunit of PI3K was one of the most upregulated proteins. EV proteomic analysis also revealed the upregulation of PPAR-α, whose effect on immune cells in the TME is often poor, ultimately leading to immunosuppression or even cancer immune escape. The role of FZD6, the other upregulated protein, in the pathogenesis of cancer is not clear. Further research is needed to investigate whether other forms of PD-L1, such as mRNAs or regulatory miRNAs, are present in EVs.

Neutrophils treated with PM-derived EVs presented high expression of the neutrophil-specific lineage marker CD66b, along with elevated levels of CXCR4 and reduced levels of CD62L. These characteristics, previously associated with aging and activation, may be indicative of a subset of neutrophils contributing to the protumoral population [46]. Researchers have shown that the lifespan of aged neutrophils is extended through the downregulation of pro-apoptotic genes and the upregulation of anti-apoptotic genes. Additionally, in aged neutrophils, the expression of adhesion-related proteins is increased. Furthermore, aged neutrophils show enhanced capacities for angiogenesis and the recruitment of immunosuppressive cells [47]. Additional investigations are needed to establish a standardized classification of neutrophil subsets.

A previous study demonstrated that subtypes of EVs, specifically 10 K, 18 K, and 100 K, which are isolated from different PM cell lines, contain MSLN [48]. Here, we report that EVs isolated from PM cells can transfer MSLN from tumor cells to the surface of neutrophils. Moreover, proteomic analysis of isolated EVs revealed an increase in MSLN levels in EVs from EPZ-6438-treated cells. MSLN is an immunogenic glycoprotein highly expressed in ovarian cancer, non-small cell lung cancer (NSCLC), and mesothelioma [49]. Its low expression in normal mesothelial cells makes MSLN a promising candidate for targeted immunotherapy in mesothelioma patients. Clinical trials are currently evaluating MSLN-targeted therapies, including combination therapies with MSLN-specific CAR-T-cell therapy and anti-PD1 therapy, which have shown promising antitumor activity in PM [50]. In addition, certain antibody–drug conjugates have demonstrated manageable safety profiles and promising antitumor effects in MSLN-positive solid tumors [51].

This study provides the first evidence that MSLN can be transferred from tumor cells to immune cells through EVs (Figure 5). Further confirmation of these data in tissue biopsies is warranted, as in vivo confirmation could have implications for the response of PM patients to therapies targeting MSLN.

## 4. Materials and Methods

### 4.1. Reagents and Antibodies

Culture media, sera, and antibiotics were obtained from Thermo Fisher Scientific (Waltham, MA, USA). The EZH2-selective inhibitor EPZ-6438 was purchased from Selleckchem (Houston, TX, USA). Anti-tubulin, anti-mouse and anti-rabbit IgG peroxidase- or FITC-conjugated antibodies and chemical reagents were obtained from Merck KGaA (Darmstadt, Germany). ECL, nitrocellulose, and protein assay kit were obtained from Bio-Rad (Hercules, CA, USA). The polyclonal antibody specific for histone H3 trimethyl lysine 27 (H3K27me3) was purchased from Active Motif (Carlsbad, CA, USA) while the monoclonal antibody specific for histone H3 was from Cell Signaling Technology (Danvers, MA, USA). The polyclonal antibodies specific for CD63 (PA5-92370), RAB27b (PA5-54096) and beta-Tubulin (PA5-16863) were from Thermo Fisher Scientific. Anti-PD-L1 (clone MIH2), anti-CXCR4 (clone QA18A64), anti-CD62L (clone W21031N), and anti-CD66b (clone G10F5) antibodies were obtained from BD Biosciences (Franklin Lakes, NJ, USA) while the anti-MSLN polyclonal antibody was purchased from Bioss (Boston, MA, USA).

### 4.2. Multicellular Spheroids

The biphasic PM-derived MSTO-211H cell line was obtained from the Istituto Scientifico Tumori (IST) cell bank in Genoa, Italy. The cells were grown under standard conditions in RPMI medium supplemented with 10% FBS, 100 μg/mL streptomycin, and 10 μg/mL penicillin at 37 °C in a humidified environment containing 5% CO_2_. Mycoplasma infection was excluded via the use of the Mycoplasma PlusTM PCR Primer Set Kit from Stratagene (La Jolla CA, USA). Multicellular spheroids were generated in nonadsorbent round-bottomed 96-well plates as previously described [14]. The 96-well plates were coated with a 1:24 dilution of polyHEMA (120 mg/mL) in 95% ethanol and dried at 37 °C for 24 h. Before use, the plates were sterilized by UV light for 30 min. For the generation of multicellular spheroids, 1 × 10^4^ cells were added to each well of a polyHEMA-coated 96-well plate and placed in a 37 °C humidified incubator with 5% CO_2_.

### 4.3. Cell Lysis and Immunoblotting

The cells were extracted with 1% NP-40 lysis buffer (50 mM Tris-HCl, pH 8.5, containing 1% NP-40, 150 mM NaCl, 10 mM EDTA, 10 mM NaF, 10 mM Na_4_P_2_O_7,_ and 0.4 mM Na_3_VO_4_) with freshly added protease inhibitors (10 μg/mL leupeptin, 4 μg/mL pepstatin and 0.1 unit/mL aprotinin). The lysates were centrifuged at 13,000× *g* for 10 min at 4 °C, and the supernatants were collected and assayed for protein concentration via the Bradford assay method (Bio-Rad, Hercules, CA, USA). Histones were acid extracted from nuclei with 0.4 N HCl and precipitated with trichloroacetic acid (TCA), followed by washing with ice-cold acetone containing 0.006% HCl and then with pure ice-cold acetone. The resulting pellets were air-dried and dissolved in a minimal volume of sterile distilled water, after which the protein concentration was determined [14]. Proteins were separated by SDS–PAGE under reducing conditions. Following SDS–PAGE, the proteins were transferred to nitrocellulose membranes, incubated with specific antibodies, and then detected with peroxidase-conjugated secondary antibodies and a chemiluminescent ECL reagent. Digital images were taken with the Bio-Rad ChemiDoc^TM^ Touch Imaging System and quantified via the Bio-Rad Image Lab 5.2.1 (Bio-Rad, Hercules, CA, USA).

### 4.4. Collection of EVs from MSTO-211H Spheroids

MSTO-211H spheroids were formed through forced aggregation in nonadherent 96-well microplates in complete medium for 24 h; subsequently, the medium was replaced with serum-free medium containing or not containing 10 μM EPZ-6438. Conditioned media from both control and treated MCSs were then collected twice daily for three days. EVs were isolated from the conditioned media via tangential flow filtration and then freeze-dried according to the procedures reported previously [28]. Tangential flow filtration was performed via a 5 kDa molecular weight cutoff filtration module; all the samples were first concentrated to a final volume of 20 mL and then diafiltered with sterile deionized water to discharge the contaminants.

### 4.5. Lyophilization of EVs

The collected EVs were aliquoted and subjected to lyophilization (T = −50 °C, *p* = 8 × 10^−1^ mbar for 72 h; Epsilon 2-6D LSCplus, Martin Christ GmbH, Osterode am Harz, Germany) using 0.5% *w*/*v* mannitol as a cryoprotectant and stored at 4 °C until further use. Before use, lyophilized EVs were rehydrated with culture media at the appropriate concentration and used immediately.

### 4.6. Determination of the Particle Size Distribution

Fresh EVs were characterized in terms of particle size distribution via a NanoSight NS300 (Malvern Instruments, Malvern, UK); each sample was analyzed at room temperature in triplicate (5 curves of 90 s each for each analysis), and the raw data were processed via NTA software v3.2 (Malvern Instruments, Malvern, UK).

### 4.7. Nile Red Staining

The amount of EVs collected from fresh samples was estimated by measuring the lipid concentration following the protocol described in [28].

### 4.8. Neutrophil/CD3+ Lymphocyte Isolation

Neutrophils and PBMCs were isolated using a density gradient protocol from buffy coats. Each buffy coat used for research was obtained from a voluntary blood donor who had signed informed consent permitting the use for research purposes. Briefly, blood was diluted 1:1 with PBS, mixed with 3% Dextran 500 in 0.9% NaCl solution at a 1:2 ratio and left standing for 30 min at room temperature in the dark to allow red blood cell sedimentation. The leukocyte-rich supernatant was collected, centrifuged, suspended in PBS, layered over fill-paque plus and then centrifuged at 1800 rpm for 15 min without a break. After centrifugation, the PBMC layer was harvested via gentle aspiration and transferred to a clean tube, while the pellet was resuspended in 0.2% NaCl for 30 s and then in 1.6% NaCl for 30 s to lyse red blood cells. The neutrophils were pelleted, washed and suspended in RPMI-1640 supplemented with 100 IU/mL penicillin, 0.1 mg/mL streptomycin and 0.25 μg/mL kanamycin. PBMCs were washed, and CD3 was isolated by using anti-CD3 (Miltenyi Biotechnology; Bergisch Gladbach, Germany) magnetic beads [52]. Before each experiment, buffy-coat isolated populations were assessed for neutrophil purity. Resuspended population was stained with anti-human CD66b a classical neutrophil marker and analyzed by FACS. The stained population was initially resolved by physical (FSC/SSC) parameters. The gated populations were evaluated for CD66b expression. We considered neutrophil population eligible for experiments when CD66b+ cells were greater than 95%.

### 4.9. Flow Cytometry Analysis

Neutrophils were stimulated with 200 μg of EVs from the supernatants of PM MCSs for 3 h. After stimulation, the neutrophils were harvested, washed, labeled with anti-CD66b, anti-PDL1, anti-MSLN, anti-CXCR4 and anti-CD62L mAbs and analyzed on an Accuri C6 plus flow cytometer (BD Biosciences). Nonspecific background fluorescence was evaluated with the appropriate isotype-matched control mAbs. Cells were gated based on CD66b expression and neutrophil markers percentage or MFI levels were evaluated on CD66^+^ population. The expression levels of the proteins were expressed as the MFI via FACSDiva software 8.0 (BD Biosciences).

### 4.10. Coculture of Neutrophils with T Cells and PM Multicellular Spheroids

Isolated peripheral CD3^+^ T cells were labeled with carboxyfluorescein succinimidyl ester (CFSE) (Stem Cell Technology; Vancouver, BC, Canada) [53] and cocultured with neutrophils incubated with EVs from the supernatants of PM MCSs for 3 h at a 3:1 ratio in 200 μL of RPMI-1640 complete medium supplemented with 5 μg/mL phytohemagglutinin (PHA). After 5 days of coculture, the CFSE dilution was analyzed via flow cytometry. After 5 days of co-culture, cells were harvested stained with PerCP-conjugated anti-CD3 and analyzed via flow cytometry. Populations were resolved and gated according to FSC/SSC parameters. The identified populations were analyzed for the CD3 expression and CFSE dilution was evaluated in CD3+ cells.

Neutrophils, either naïve, treated with LPS (10 μg/mL) or challenged (3 h) with EVs isolated from the conditioned media of MSTO-211H spheroids untreated or treated with EPZ-6438, were added to MSTO-211H spheroids and incubated for 48 h. Subsequently, MSTO-211H spheroids were disaggregated and viable cells were counted using a Burker chamber (Merck KGaA; Darmstadt, Germany).

### 4.11. Statistical Analysis

The data are expressed as the means ± SDs. The statistical significance of differences between the two groups was determined by a two-tailed Student’s *t* test. The significance of differences among multiple groups was determined by one-way ANOVA. All experiments were performed at least in triplicate (*n* = 3). *p* ≤ 0.05 was considered statistically significant. All the statistical analyses were performed via GraphPad Prism 8 (GraphPad Software, La Jolla, CA, USA).

### 4.12. Proteomic Analysis and Data Processing

#### 4.12.1. In-Solution Digestion

Prior to proteomic analysis, protein digestion was performed with trypsin for both the cells and the extracellular vesicles. Briefly, samples containing 100 μg of protein in 25 μL of 100 mM NH_4_HCO_3_ were prepared. A total of 2.5 μL of 200 mM DTT (Sigma–Aldrich) was used to reduce the proteins, which were then incubated at 90 °C for 20 min. The following alkylation was executed with 10 μL of 200 mM iodoacetamide (Sigma–Aldrich) for 1 h at room temperature in the dark, and the excess iodoacetamide was removed with 200 mM DTT. Three hundred microlitres of Milli-Q water and 100 μL of NH_4_HCO_3_ were added to dilute and increase the pH to 7.5–8.0, and 5 μg of trypsin (Promega, Madison, WI, USA) was added to digest the proteins O/N at 37 °C. Trypsin activity was stopped by the addition of 2 μL of neat formic acid, and the digests were dried with a speed vacuum. The peptide digests were desalted on a Discovery^®^ DSC-18 solid phase extraction 96-well plate (25 mg/well) (Merck KGaA) as reported elsewhere.

#### 4.12.2. Mass Spectrometry Analysis of Cell Lysates

LC–MS/MS analyses were performed via a micro-LC Eksigent Technologies (Dublin, OH, USA) system with a stationary phase Halo Fused C18 column (0.5 × 100 mm, 2.7 μm; Eksigent Technologies, Dublin, OH, USA). The oven temperature was set to 40 °C, and the injection volume was 4 μL. The mobile phase was a mixture of 0.1% (*v*/*v*) formic acid in water (A) and 0.1% (*v*/*v*) formic acid in acetonitrile (B). The flow rate of the elution was 15.0 μL/min, with an increasing concentration of solvent B from 2% to 40% in 30 min. The chromatographic system was interfaced with a 5600+ TripleTOF system equipped with a DuoSpray Ion Source and CDS (Calibrant Delivery System) (SCIEX, Concord, ON, Canada). The samples used to generate the SWATH-MS spectral library were subjected to traditional data-dependent acquisition (DDA) and to cyclic data-independent analysis (DIA) of the mass spectra via a 25-Da window. The mass spectrometer analysis was performed in positive ionization mode using a mass range of 100–1500 Da (TOF scan with an accumulation time of 0.25 s), followed by an MS/MS product ion scan from 200 to 1250 Da (accumulation time of 5.0 ms) with the abundance threshold set at 30 cps (35 candidate ions can be monitored during every cycle). The samples were then subjected to cyclic data independent analysis (DIA) of the mass spectra via a 25-Da window: the mass spectrometer was operated such that a 50 ms survey scan (TOF-MS) was performed, and subsequent MS/MS experiments were performed on all the precursors. These MS/MS experiments were performed in a cyclic manner using an accumulation time of 40 ms per 25-Da swath (36 swaths in total) for a total cycle time of 1.5408 s. The ions were fragmented for each MS/MS experiment in the collision cell using the rolling collision energy. Analyst TF 1.7 (SCIEX, Concord, ON, Canada) was used to acquire MS data, and three instrumental replicates for each sample were subjected to DIA)

Protein identification was performed via Protein Pilot v. 4.2 (SCIEX) and Mascot v. 2.4 (Matrix Science, London, UK). For Protein Pilot, the following parameters were chosen to search the MS files: cysteine alkylation, trypsin digestion, no special factors, and a false discovery rate of 1%. Additionally, Mascot was employed with the following parameters: trypsin as the enzyme, 2 missed cleavages allowed, a peptide mass tolerance of 50 ppm, and an MS/MS tolerance of 0.1 Da. Peptide charges of 2+, 3+, and 4+ were included in the search, and the monoisotopic mass option was selected. The instrument used for the experiments was an ESI-QUAD-TOF instrument. The specified modifications for the search included fixed modifications for carbamidomethyl cysteines and oxidized methionine as a variable modification. The UniProt/Swiss-Prot reviewed database containing human protein (UniProt, version 01042019) was utilized as the reference database for the searches. For protein quantification, an ion chromatogram of all unique ions for a given peptide was integrated to obtain label-free quantification via PeakView 2.0 and MarkerView 1.2. (Sciex, Berlin, Germany). An integrated assay library was built with the DDA results and a protein FDR threshold of 1%. Six transitions per peptide and six peptides per protein were extracted from the SWATH files, excluding peptides with modifications as well as shared peptides. A *t* test was eventually performed on peptides with a false discovery rate < 1% exported in MarkerView. A *p* value < 0.05, as well as a fold change > 1.3, was selected as the maximum value to choose proteins with lower or higher abundance.

### 4.13. Mass Spectrometry Analysis of Extracellular Vesicles

The digested peptides were analyzed on an Ultimate 3000 RSLC coupled directly to an Orbitrap Exploris 480 with a high-field asymmetric waveform ion mobility spectrometry system (FAIMSpro) (all Thermo Fisher Scientific). The samples were injected into a reversed-phase C18 column (15 cm × 75 µm i.d., Thermo Fisher Scientific) and eluted with a gradient of 6% to 95% mobile phase B over 41 min by applying a flow rate of 500 nL/min, followed by equilibration with 6% mobile phase B for 1 min. The acquisition time of one sample was 41 min, and the total recording of the MS spectra was carried out at positive resolution with a high voltage of 2500 V. The FAIMS interface was at standard resolution with a CV of −45 V. The acquisition was performed in data-independent mode (DIA): the precursor mass range was set between 400 and 900, the isolation window was 8 *m*/*z*, the window overlap was 1 *m*/*z*, the HCD collision energy was 27%, the orbitrap resolution was 30,000, and the RF lens was 50%. The normalized AGC target was set to 1000, the maximum injection time was 25 ms, and the microscan was 1. For DIA data processing, DIA-NN (version 1.8.1) was used: identification was performed with “library-free search” and “deep learning-based spectra, RTs and IMs prediction” enabled. The enzyme was set to trypsin/P, precursors of charge states 1–4, peptide lengths 7–30, and precursor *m*/*z* 400–900 were considered with a maximum of two missed cleavages. Carbamidomethylation on C was set as a fixed modification, and oxidation on M was set as a variable modification, with a maximum of two variable modifications per peptide. A human UniProt protein database was used (downloaded on 5 May 2024). The FDR was set to 1%. Statistical analyses and *t* tests were performed on protein abundances via Excel.

## 5. Conclusions

While this study was limited to a single PM cell line, MSTO-211H of the mixed histotype, which may constrain the generalizability of the findings to other mesothelioma subtypes, the results nonetheless offer valuable insights. Importantly, they underscore the potential translational significance of this work and draw attention to possible unintended consequences of therapeutic strategies targeting EZH2, warranting further investigation across additional models.

In detail, our findings reveal that treatment with tazemetostat significantly increases the release of EVs from PM cells. When we investigated the effects of tumor-derived EVs on naïve neutrophils, we observed that even a short exposure to EVs from tazemetostat-treated cells prompted a shift in these neutrophils toward a protumor phenotype, marked by elevated levels of PD-L1 and MSLN on their surface. Notably, our study is the first to illustrate that PM-derived EVs can promote the development of immunosuppressive neutrophils, ultimately inhibiting T-cell immunity. These findings underscore the critical role of EVs in fostering an immunosuppressive microenvironment and enhancing our understanding of neutrophil biology and function in PM.

Additionally, proteomic analysis of isolated EVs indicated an increase in MSLN levels in those derived from tazemetostat-treated cells. MSLN is a highly immunogenic glycoprotein predominantly expressed in PM, with minimal expression in normal mesothelial cells, positioning it as a promising target for PM management. Importantly, our study provides the first evidence that MSLN can be transferred from tumor cells to immune cells via EVs. Further investigation of these findings in tissue biopsies is necessary, as in vivo validation could significantly impact how PM patients respond to MSLN-targeted therapies.

## Data Availability

The authors declare that the data supporting the findings of this study are available within the paper and its Supplementary Information files. Should any raw data files be needed in another format, they are available from the corresponding author upon reasonable request. The mass spectrometry proteomics data have been deposited to the ProteomeXchange Consortium via the PRIDE partner repository (PXD057566).

## Supplementary Materials

The supporting information can be downloaded at: https://www.mdpi.com/article/10.3390/ijms262110328/s1.

## References

[1] Thives L.P., Ghisi E., Júnior J.J.T., Vieira A.S. (2022). Is asbestos still a problem in the world? A current review. J. Environ. Manag..

[2] Popat S., Baas P., Faivre-Finn C., Girard N., Nicholson A.G., Nowak A.K., Opitz I., Scherpereel A., Reck M., ESMO Guidelines Committee (2022). Malignant pleural mesothelioma: ESMO Clinical Practice Guidelines for diagnosis, treatment and follow-up. Ann. Oncol..

[3] Vogelzang N.J., Rusthoven J.J., Symanowski J., Denham C., Kaukel E., Ruffie P., Gatzemeier U., Boyer M., Emri S., Manegold C. (2003). Phase III study of pemetrexed in combination with cisplatin versus cisplatin alone in patients with malignant pleural mesothelioma. J. Clin. Oncol..

[4] Baas P., Scherpereel A., Nowak A.K., Fujimoto N., Peters S., Tsao A.S., Mansfield A.S., Popat S., Jahan T., Antonia S. (2021). First-line nivolumab plus ipilimumab in unresectable malignant pleural mesothelioma (CheckMate 743): A multicentre, randomised, open-label, phase 3 trial. Lancet.

[5] Chu Q., Perrone F., Greillier L., Tu W., Piccirillo M.C., Grosso F., Russo G.L., Florescu M., Mencoboni M., Morabito A. (2023). Pembrolizumab plus chemotherapy versus chemotherapy in untreated advanced pleural mesothelioma in Canada, Italy, and France: A phase 3, open-label, randomised controlled trial. Lancet.

[6] Gray S.G., Meirson T., Mutti L. (2024). Based on the Real-World Results From Australia, Immunotherapy Is Not a Good Option for Patients with Mesothelioma. J. Thorac. Oncol..

[7] Jin L., Gu W., Li X., Xie L., Wang L., Chen Z. (2020). PD-L1 and prognosis in patients with malignant pleural mesothelioma: A meta-analysis and bioinformatics study. Ther. Adv. Med. Oncol..

[8] Cedres S., Valdivia A., Iranzo P., Callejo A., Pardo N., Navarro A., Martinez-Marti A., Assaf-Pastrana J.D., Felip E., Garrido P. (2023). Current State-of-the-Art Therapy for Malignant Pleural Mesothelioma and Future Options Centered on Immunotherapy. Cancers.

[9] KaKaplan M.A., Şendur M.A.N., Cangır A.K., Fırat P., Göker E., Kılıçkap S., Oyan B., Öz A.B., Özdemir F., Özyiğit G. (2023). Established and new treatment roadmaps for pleural mesothelioma: Opinions of the Turkish Collaborative Group. Curr. Probl. Cancer.

[10] Vincenzi F., Rotondo J.C., Pasquini S., Di Virgilio F., Varani K., Tognon M. (2021). A3 Adenosine and P2X7 Purinergic Receptors as New Targets for an Innovative Pharmacological Therapy of Malignant Pleural Mesothelioma. Front. Oncol..

[11] Baldini E.H., Richards W.G., Gill R.R., Goodman B.M., Winfrey O.K., Eisen H.M., Mak R.H., Chen A.B., Kozono D.E., Bueno R. (2015). Updated patterns of failure after multimodality therapy for malignant pleural mesothelioma. J. Thorac. Cardiovasc. Surg..

[12] Zauderer M.G., Szlosarek P.W., Le Moulec S., Popat S., Taylor P., Planchard D., Scherpereel A., Koczywas M., Forster M., Cameron R.B. (2022). EZH2 inhibitor tazemetostat in patients with relapsed or refractory, BAP1-inactivated malignant pleural mesothelioma: A multicentre, open-label, phase 2 study. Lancet Oncol..

[13] Shi Y., Wang X.X., Zhuang Y.W., Jiang Y., Melcher K., Xu H.E. (2017). Structure of the PRC2 complex and application to drug discovery. Acta Pharmacol. Sin..

[14] Pinton G., Wang Z., Balzano C., Missaglia S., Tavian D., Boldorini R., Fennell D.A., Griffin M., Moro L. (2021). CDKN2A Determines Mesothelioma Cell Fate to EZH2 Inhibition. Front. Oncol..

[15] Mola S., Pinton G., Erreni M., Corazzari M., De Andrea M., Grolla A.A., Martini V., Moro L., Porta C. (2021). Inhibition of the Histone Methyltransferase EZH2 Enhances Protumor Monocyte Recruitment in Human Mesothelioma Spheroids. Int. J. Mol. Sci..

[16] Takasugi M., Yoshida Y., Ohtani N. (2022). Cellular senescence and the tumour microenvironment. Mol. Oncol..

[17] Faget D.V., Ren Q., Stewart S.A. (2019). Unmasking senescence: Context-dependent effects of SASP in cancer. Nat. Rev. Cancer.

[18] Kalluri R., McAndrews K.M. (2023). The role of extracellular vesicles in cancer. Cell.

[19] Kalluri R., LeBleu V.S. (2020). The biology, function, and biomedical applications of exosomes. Science.

[20] Ma F., Vayalil J., Lee G., Wang Y., Peng G. (2021). Emerging role of tumor-derived extracellular vesicles in T-cell suppression and dysfunction in the tumor microenvironment. J. Immunother. Cancer.

[21] Stella G.M., Marchiò C., Bari E., Ferrarotti I., Bertuccio F.R., Di Gennaro A., Abbott D.M., Putignano P., Campo I., Torre M.L. (2023). The Genes-Stemness-Secretome Interplay in Malignant Pleural Mesothelioma: Molecular Dynamics and Clinical Hints. Int. J. Mol. Sci..

[22] de Miguel-Perez D., Russo A., Arrieta O., Ak M., Barron F., Gunasekaran M., Mamindla P., Lara-Mejia L., Peterson C.B., Er M.E. (2022). Extracellular vesicle PD-L1 dynamics predict durable response to immune-checkpoint inhibitors and survival in patients with non-small cell lung cancer. J. Exp. Clin. Cancer Res..

[23] Yu Z.L., Liu J.Y., Chen G. (2022). Small extracellular vesicle PD-L1 in cancer: The knowns and unknowns. NPJ Precis. Oncol..

[24] Mukaida N., Sasaki S.-I., Baba T. (2020). Two-Faced Roles of Tumor-Associated Neutrophils in Cancer Development and Progression. Int. J. Mol. Sci..

[25] Liu S., Wu W., Du Y., Yin H., Chen Q., Yu W., Wang W., Yu J., Liu L., Lou W. (2023). The evolution and heterogeneity of neutrophils in cancers: Origins, subsets, functions, orchestrations and clinical applications. Mol. Cancer.

[26] Zilionis R., Engblom C., Pfirschke C., Savova V., Zemmour D., Saatcioglu H.D., Krishnan I., Maroni G., Meyerovitz C.V., Kerwin C.M. (2019). Single-Cell transcriptomics of human and mouse lung cancers reveals conserved myeloid populations across individuals and species. Immunity.

[27] Okita R., Kawamoto N., Okada M., Inokawa H., Yamamoto N., Murakami T., Ikeda E. (2023). Preoperative neutrophil-to-lymphocyte ratio correlates with PD-L1 expression in immune cells of patients with malignant pleural mesothelioma and predicts prognosis. Sci. Rep..

[28] Bari E., Perteghella S., Di Silvestre D., Sorlini M., Catenacci L., Sorrenti M., Marrubini G., Rossi R., Tripodo G., Mauri P. (2018). Pilot Production of Mesenchymal Stem/Stromal Freeze-Dried Secretome for Cell-Free Regenerative Nanomedicine: A Validated GMP-Compliant Process. Cells.

[29] Clancy J.W., D’SOuza-Schorey C. (2023). Tumor-Derived Extracellular Vesicles: Multifunctional Entities in the Tumor Microenvironment. Annu. Rev. Pathol..

[30] Giese M.A., Hind L.E., Huttenlocher A. (2019). Neutrophil plasticity in the tumor microenvironment. Blood.

[31] Sabour-Takanlou M., Sabour-Takanlou L., Biray-Avci C. (2024). EZH2-associated tumor malignancy: A prominent target for cancer treatment. Clin. Genet..

[32] Gao M., Li Y., Cao P., Liu H., Chen J., Kang S. (2023). Exploring the therapeutic potential of targeting polycomb repressive complex 2 in lung cancer. Front. Oncol..

[33] Straining R., Eighmy W. (2022). Tazemetostat: EZH2 Inhibitor. J. Adv. Pract. Oncol..

[34] Kang N., Eccleston M., Clermont P.L., Latarani M., Male D.K., Wang Y., Crea F. (2020). EZH2 inhibition: A promising strategy to prevent cancer immune editing. Epigenomics.

[35] Sun S., Yu F., Xu D., Zheng H., Li M. (2022). EZH2, a prominent orchestrator of genetic and epigenetic regulation of solid tumor microenvironment and immunotherapy. Biochim. Biophys. Acta Rev. Cancer.

[36] Parvez A., Choudhary F., Mudgal P., Khan R., Qureshi K.A., Farooqi H., Aspatwar A. (2023). PD-1 and PD-L1: Architects of immune symphony and immunotherapy breakthroughs in cancer treatment. Front. Immunol..

[37] Gong Y.T., Zhang L.J., Liu Y.C., Tang M., Lin J.Y., Chen X.Y., Chen Y.X., Yan Y., Zhang W.D., Jin J.M. (2023). Neutrophils as potential therapeutic targets for breast cancer. Pharmacol. Res..

[38] Kumar M.A., Baba S.K., Sadida H.Q., Marzooqi S.A., Jerobin J., Altemani F.H., Algehainy N., Alanazi M.A., Abou-Samra A.B., Kumar R. (2024). Extracellular vesicles as tools and targets in therapy for diseases. Signal Transduct. Target. Ther..

[39] Thakur A., Wei Z., Chen H.J. (2023). Editorial: Extracellular vesicles and cell–cell communication in normal cellular processes and cancer. Front. Mol. Biosci..

[40] Balkwill F.R., Capasso M., Hagemann T. (2012). The tumor microenvironment at a glance. J. Cell Sci..

[41] Bandini S., Ulivi P., Rossi T. (2024). Extracellular Vesicles, Circulating Tumor Cells, and Immune Checkpoint Inhibitors: Hints and Promises. Cells.

[42] Wang H., Ye X., Spanos M., Wang H., Yang Z., Li G., Xiao J., Zhou L. (2023). Exosomal Non-Coding RNA Mediates Macrophage Polarization: Roles in Cardiovascular Diseases. Biology.

[43] Shi Y., Zhang J., Mao Z., Jiang H., Liu W., Shi H., Ji R., Xu W., Qian H., Zhang X. (2020). Extracellular Vesicles From Gastric Cancer Cells Induce PD-L1 Expression on Neutrophils to Suppress T-Cell Immunity. Front. Oncol..

[44] Gao Y., Feng Y., Liu S., Zhang Y., Wang J., Qin T., Chen P., Li K. (2023). Immune-independent acquired resistance to PD-L1 antibody initiated by PD-L1 upregulation via PI3K/AKT signaling can be reversed by anlotinib. Cancer Med..

[45] Guo F., Kong W., Li D., Zhao G., Anwar M., Xia F., Zhang Y., Ma C., Ma X. (2024). M2-type tumor-associated macrophages upregulated PD-L1 expression in cervical cancer via the PI3K/AKT pathway. Eur. J. Med. Res..

[46] Peng Z., Liu C., Victor A.R., Cao D.Y., Veiras L.C., Bernstein E.A., Khan Z., Giani J.F., Cui X., Bernstein K.E. (2021). Tumors exploit CXCR4hiCD62Llo aged neutrophils to facilitate metastatic spread. Oncoimmunology.

[47] Yang C., Wang Z., Li L., Zhang Z., Jin X., Wu P., Sun S., Pan J., Su K., Jia F. (2021). Aged neutrophils form mitochondria-dependent vital NETs to promote breast cancer lung metastasis. J. Immunother. Cancer.

[48] Ahmadzada T., Vijayan A., Vafaee F., Azimi A., Reid G., Clarke S., Kao S., Grau G.E., Hosseini-Beheshti E. (2023). Small and Large Extracellular Vesicles Derived from Pleural Mesothelioma Cell Lines Offer Biomarker Potential. Cancers.

[49] Ye L., Lou Y., Lu L., Fan X. (2019). Mesothelin-targeted second generation CAR-T cells inhibit growth of mesothelin-expressing tumors in vivo. Exp. Ther. Med..

[50] Yun K.M., Bazhenova L. (2024). Emerging New Targets in Systemic Therapy for Malignant Pleural Mesothelioma. Cancers.

[51] Sun Z., Chu X., Adams C., Ilina T.V., Guerrero M., Lin G., Chen C., Jelev D., Ishima R., Li W. (2023). Preclinical assessment of a novel human antibody VH domain targeting mesothelin as an antibody-drug conjugate. Mol. Ther. Oncolytics.

[52] Pitti R.M., Marsters S.A., Lawrence D.A., Roy M., Kischkel F.C., Dowd P., Huang A., Donahue C.J., Sherwood S.W., Baldwin D.T. (1998). Genomic amplification of a decoy receptor for Fas ligand in lung and colon cancer. Nature.

[53] Lyons A.B., Parish C.R. (1994). Determination of lymphocyte division by flow cytometry. J. Immunol. Methods.

