# Supplementary material for: EZH2 Inhibition in Mesothelioma Cells Increases the Release of Extracellular Vesicles That Skew Neutrophils Toward a Protumor Phenotype
Source: Int J Mol Sci. 2025 Oct 23;26(21):10328. doi: 10.3390/ijms262110328 (PMC12607341; doi:10.3390/ijms262110328)
Supplement: Supplementary file 1 [file ijms-26-10328-s001.zip › ijms-3885386-supplementary.pdf]

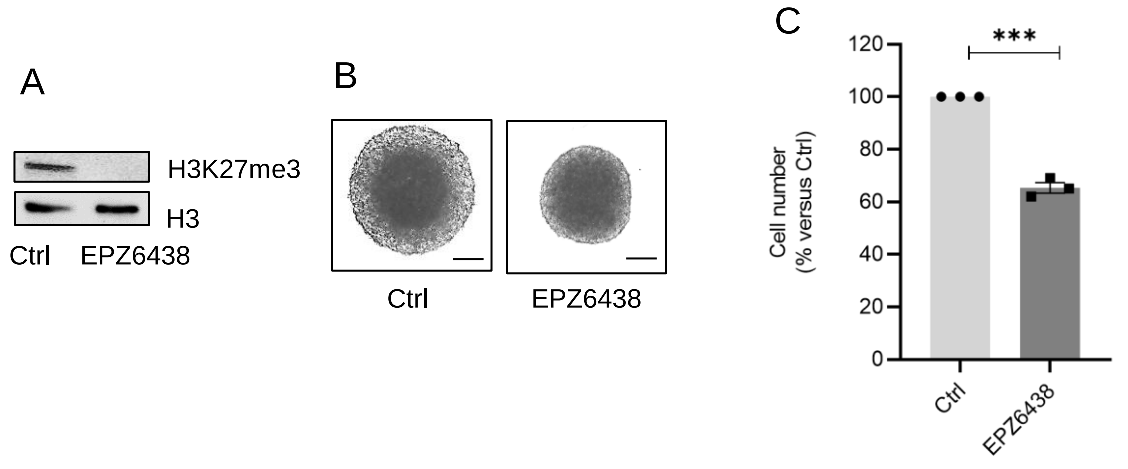


**Figure S1** (**A**) Representative Western blot analysis of H3K27me3 in MSTO-211H cells cultured as MCSs ± 72 hours treatment with EPZ-6438. Histone H3 was used as the loading control. (**B**) Representative phase contrast images (×40 magnification) of MSTO-211H cultured as MCSs ± 72 hours treatment with EPZ-6438 Scale bar 100 µm. (**C**) Bar graph shows the percentage of viable MSTO-211H cells cultured as MCSs ± 72 hours treatment with EPZ-6438. Each bar represents the mean of three independent experiments ± SD,SD,***p≤ 0.005.

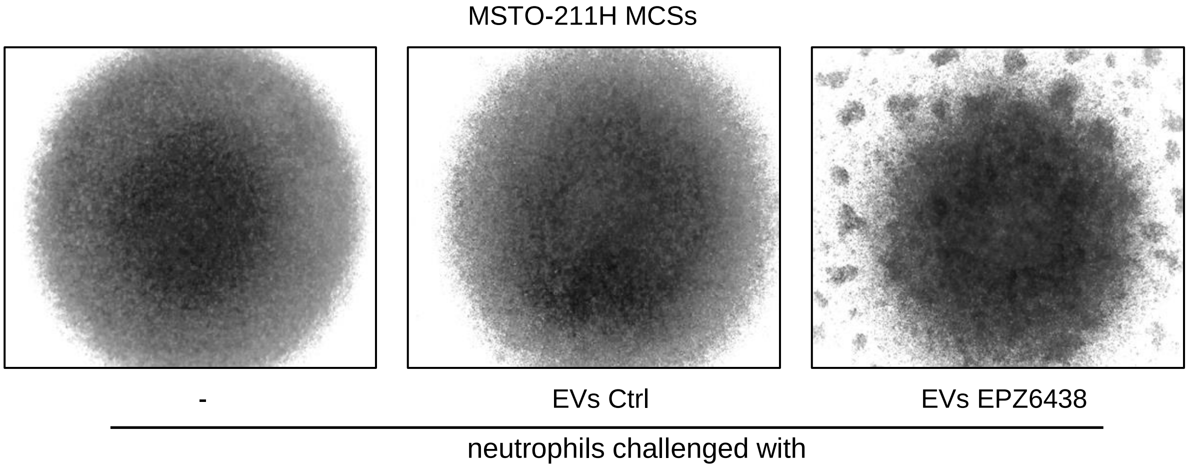


**Figure S2** Representative phase contrast images (×40 magnification) of neutrophils incubated for 3 hours in a 96-well plate with EVs isolated from MSTO-211H MCSs ± 72 hours of treatment with EPZ-6438.

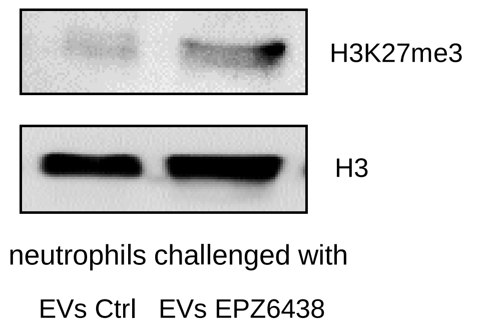


**Figure S3** Representative Western blot analysis of H3K27me3 in neutrophils challenged 3 hours with EVs from MSTO-211H MCSs untreated or treated with EPZ-6438. Histone H3 was used as the loading control.
